# Genetic Evidence for a Link Between Glycolysis and DNA Replication

**DOI:** 10.1371/journal.pone.0000447

**Published:** 2007-05-16

**Authors:** Laurent Jannière, Danielle Canceill, Catherine Suski, Sophie Kanga, Bérengère Dalmais, Roxane Lestini, Anne-Françoise Monnier, Jérôme Chapuis, Alexander Bolotin, Marina Titok, Emmanuelle Le Chatelier, S. Dusko Ehrlich

**Affiliations:** Laboratoire de Génétique Microbienne, INRA, Jouy en Josas, France; University of Massachusetts, United States of America

## Abstract

**Background:**

A challenging goal in biology is to understand how the principal cellular functions are integrated so that cells achieve viability and optimal fitness in a wide range of nutritional conditions.

**Methodology/Principal Findings:**

We report here a tight link between glycolysis and DNA synthesis. The link, discovered during an analysis of suppressors of thermosensitive replication mutants in bacterium *Bacillus subtilis*, is very strong as some metabolic alterations fully restore viability to replication mutants in which a lethal arrest of DNA synthesis otherwise occurs at a high, restrictive, temperature. Full restoration of viability by such alterations was limited to cells with mutations in three elongation factors (the lagging strand DnaE polymerase, the primase and the helicase) out of a large set of thermosensitive mutants affected in most of the replication proteins. Restoration of viability resulted, at least in part, from maintenance of replication protein activity at high temperature. Physiological studies suggested that this restoration depended on the activity of the three-carbon part of the glycolysis/gluconeogenesis pathway and occurred in both glycolytic and gluconeogenic regimens. Restoration took place abruptly over a narrow range of expression of genes in the three-carbon part of glycolysis. However, the absolute value of this range varied greatly with the allele in question. Finally, restoration of cell viability did not appear to be the result of a decrease in growth rate or an induction of major stress responses.

**Conclusions/Significance:**

Our findings provide the first evidence for a genetic system that connects DNA chain elongation to glycolysis. Its role may be to modulate some aspect of DNA synthesis in response to the energy provided by the environment and the underlying mechanism is discussed. It is proposed that related systems are ubiquitous.

## Introduction

The replisome is a multiprotein machine that replicates DNA in living organisms [1]–[3]. In the Gram-positive bacterium *Bacillus subtilis* and related micro-organisms, this machine contains two different polymerases which are thought to be specialized in leading (PolC) and lagging (DnaE) strand polymerization [4], [5]. For processivity, the polymerases bind to a protein (the β clamp) that encircles and slides along the DNA. This protein is loaded on the duplex by proteins DnaX, δ and δ′ [6]. The duplex is melted ahead of the polymerases by the DnaC helicase assembled on DNA by the combined action of the initiation factors DnaA (or PriA), DnaB, DnaD and DnaI [7]. RNA primers used by polymerases for strand synthesis are produced by the DnaG primase. While replisome assembly at origins is tightly regulated so that DNA synthesis initiates once per cell cycle [8]–[10], its progression is generally thought to be unregulated, copying undamaged DNA from the origin to the terminus in an uncontrolled way (see however below).

Glycolysis, gluconeogenesis, the pentose phosphate pathway and the tricarboxylic acid pathway are major elements of the central carbon metabolism in which nutrients provided by the environment are converted into building blocks and used for generating energy and reducing power for biomass synthesis. Glycolysis is a nine reactions pathway that is conventionally split in two parts. In the first part, glucose 6-phosphate is converted into glyceraldehyde 3-phosphate. This set of reactions, directly fuelled by glycolytic nutrients, is efficiently shunted by the pentose phosphate pathway. In the second part, glyceraldehyde 3-phosphate is transformed into pyruvate. These reactions, termed thereafter the three-carbon part of glycolysis, are required for degradation of virtually all carbon sources and cannot be efficiently shunted in most organisms. Thus, they play a key role in cell metabolism. The gluconeogenesis pathway operates when carbon sources feed the bottom part of the central carbon metabolism. It uses most of the glycolytic reactions in the opposite direction to produce glucose 6-phosphate.

Several studies indicate that DNA replication might be linked to cell metabolism. First, the rate of replication is coupled to nutrient richness in bacteria. In *Escherichia coli*, this coupling is achieved by regulating the frequency of initiation of replication and, in slow-growing cells (generation time ≥70 mn), by modulating the rate of DNA chain elongation [11]–[13]. Second, the stringent response induced by chemicals mimicking a nutrient starvation inhibits initiation of replication in *E. coli* and arrests DNA elongation at specific sites in the chromosome of *B. subtilis* ([14], [15], reviewed in [13], [16]). This response also interferes with plasmid replication (reviewed in [17]). Third, DNA synthesis takes place in the reductive phase of a metabolic respiration/reduction cycle in *Saccharomyces cerevisiae* [18], [19]. Fourth, DNA synthesis is stimulated by glucose in SV40 and in HeLa cells grown in hypoxia [20]. Fifth, mutations in glycolytic genes encoding the enolase (termed thereafter Eno), the phosphoglycerate kinase (Pgk) or the glucokinase suppress a thermosensitive (Ts) mutation in the *S. cerevisiae* MCM1 protein [21]. This multifunctional protein, required for stable maintenance of (mini)-chromosomes, binds sequences closed to replication origins for stimulating initiation of DNA synthesis [22]–[24]. It also regulates transcription of genes involved in diverse cellular functions including replication and cell-cycle factors (see for instances [25]–[27]). Finally, stimulation of histone H2B gene expression, which is essential for S phase progression, strictly depends on the glyceraldehyde 3-P dehydrogenase glycolytic enzyme (GapA) in human cells. In this task, GapA is complexed to a transcriptional co-activator of the H2B gene (ACO-S) and is thought to regulate the activity of the co-activator by sensing the NAD/NADH redox status [28]. The H2B regulation pathway might also involve the lactate dehydrogenase (LDH) [28]. While these observations argue for a functional link between replication and metabolism, the underlying key components and mechanism remain largely unknown. The findings reported here uncover for the first time a robust metabolism/replication link in the bacterium *B. subtilis*. Key elements of the link have been identified. They are the DnaE (the lagging strand polymerase), DnaG (primase) and DnaC (helicase) replisomal enzymes and the activity of the three-carbon part of the glycolysis/gluconeogenesis pathway. The mechanism and function of the link are discussed.

## Results

### Genetic link between DNA replication and glycolysis

**Figure 1:**
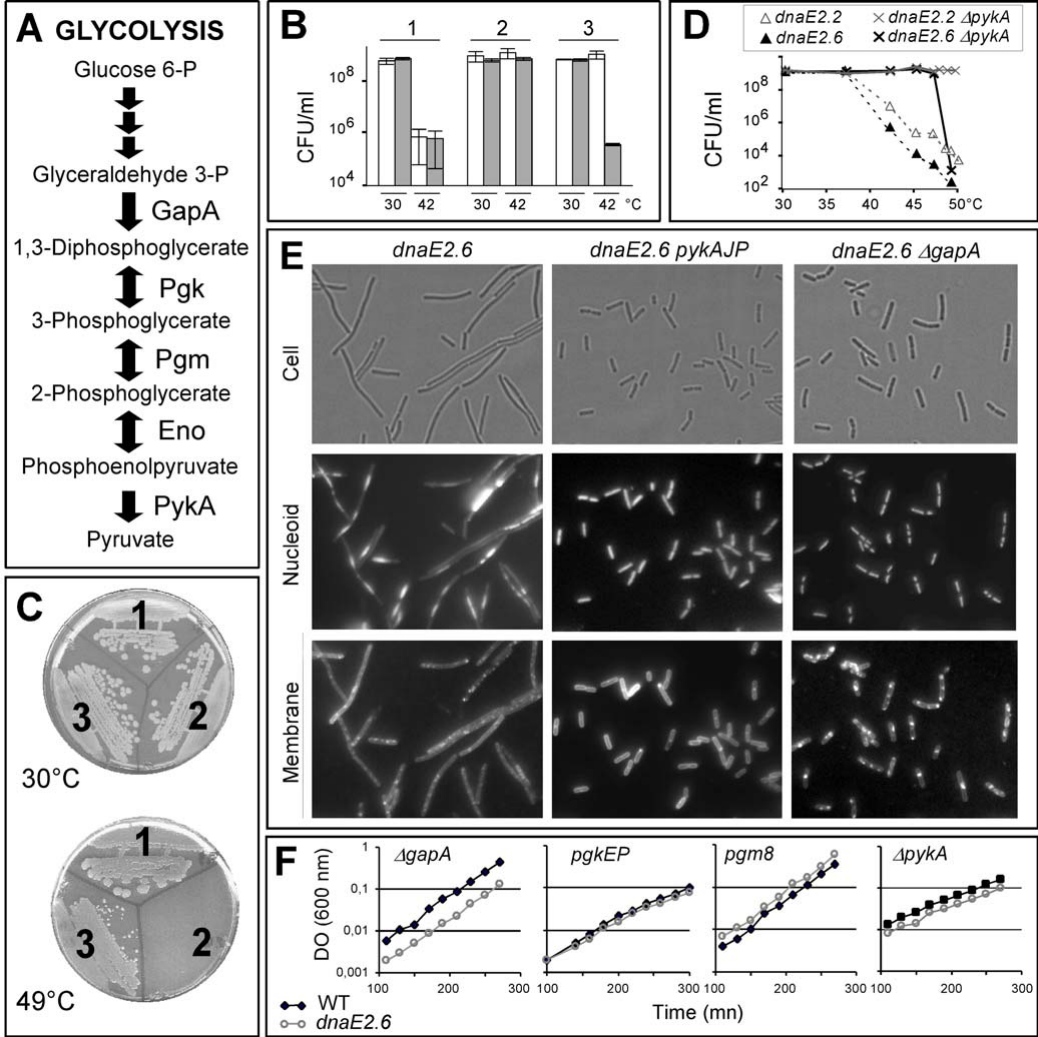
Glycolytic mutations suppress *dnaE*(Ts) mutants. (A) Schematic representation of glycolysis. GapA: glyceraldehyde 3-phosphate dehydrogenase; Pgk: phosphoglycerate kinase; Pgm: phosphoglycerate mutase; Eno: enolase; PykA: pyruvate kinase. (B) Complementation assay. The *dnaE2.6* (1), *dnaE2.6 pgm8* (2) and *dnaE2.6 pgm8 pgm^ind^* (3) strains were grown in LB at 30°C and plated on the same broth containing (grey bars) or not (white bars) 0.5% of xylose (the WT copy of *pgm* in strain 3 was expressed from a promoter induced by xylose). Upon incubation at permissive or restrictive temperatures, the concentration of cell forming unit (CFU/mL) was determined. (C–E) Growth of Ts and suppressed strains at various temperatures was analyzed by streaking (C; 1: WT TF8A strain; 2: *dnaE2.10*; 3: *dnaE2.10 pgm25*), plating (D) and optical microscopy after 2h of growth at 42°C (E). Cell pictures are as follows: cell: bright font; nucleoid: DAPI staining; membrane: FM5-95 staining. (F) Growth of four different *dnaE2.6* suppressed strains and the corresponding metabolic mutants at restrictive temperature (40°C) in LB liquid broth (followed by measurement of optical density).

To identify proteins functionally connected to the lagging strand DnaE polymerase, a search was conducted for extragenic suppressors of four *dnaE* thermosensitive (Ts) mutations (named *dnaE2.2*, *dnaE2.4*, *dnaE2.6* and *dnaE2.10*, see Table 1 for mutation details). Shortly after a shift to restrictive temperature, the Ts mutants cease (or dramatically reduce) DNA synthesis, make filaments and die [5]. Among a collection of extragenic suppressors isolated at 45–49°C on a glycolytic rich medium (LB, see Materials and Methods for details), seven were successively mapped. Surprisingly, rather than occurring in functions related to DNA metabolism, they fell into four metabolic genes (*pgk*, *pgm*, *eno* and *pykA*) ensuring the terminal reactions of glycolysis (Fig. 1A, the suppressed strains and metabolic mutations are listed Table 2). Three sets of data showed that these mutations were responsible for suppression. First, the genetic transfer of metabolic mutations (*pgkEP*, *pgm8, pgmIP* or *pykAJP*, labeled by a genetic marker) into parental *dnaE*(Ts) strains led to Tr transformants. Second, four *dnaE*(Ts) mutants carrying a suppressive mutation in *pgk*, *pgm*, *eno* or *pykA* became Ts when expressing a WT copy of the corresponding glycolytic gene from a xylose inducible promoter (as exemplified in Fig. 1B with the *pgm8* suppressor). Third, a deliberately generated deletion of *pykA* caused *dnaE*(Ts) suppression (see below).

**Table 1:** Mutations in *dnaE*(Ts) alleles

Allele	DNA mutations*	Protein mutations*
*dnaE2.2*	T(160)C	C(54)R
	T(176)C	I(59)T
	T(422)C	F(141)S
	T(672)C	-
*dnaE2.4*	A(627)G	-
	A(1067)G	Y(356)C
	A(1072)C	T(358)P
*dnaE2.6*	C(237)T	-
	T(284)C	L(95)P
	T(872)C	L(291)P
*dnaE2.10*	T(341)C	L(114)P
	A(767)G	Q(256)R
	T(976)C	W(326)R
	A(1713)G	-
	A(1880)G	D(627)G
	A(2024)C	D(675)A
	A(2123)G	D(708)G

* Numbers in brackets indicate the position of the mutation in the DNA or in the protein (numbering starts at the first base or codon of the open reading frame).

**Table 2:** Spontaneously isolated suppressive mutations

Mutated genes	Allele	*dnaE*(Ts) background§	DNA mutations*	Protein mutations*
*pgk*	*EP*	*dnaE2.4*	G(154)Δ	G(51)stop+8
*pgk*	*34*	*dnaE2.10*	ND	ND
*pgm*	*IP*	*dnaE2.4*	T(1349)G	I(450)S
*pgm*	*8*	*dnaE2.6*	C(995)T	A(332)V
*pgm*	*25*	*dnaE2.10*	A(335)G	H(112)R
*eno*	*LP*	*dnaE2.4*	G(404)A	G(135)E
*pykA*	*JP*	*dnaE2.4*	in frame Δ(622–702)	Δ(208–234)

§ The listed metabolic mutations were initially isolated in the indicated *dnaE*(Ts) background.

* Numbers in brackets indicate the position of the mutation in the DNA or in the protein (numbering starts at the first base or codon of the open reading frame). Δ stands for deletion. ND: not determined. Stop+8: Stop codon 8 residues downstream of G51.

Three sets of data demonstrated that glycolytic mutations are strong *dnaE*(Ts) suppressors. First, the seven suppressed strains carrying a mapped suppressive mutation grew vigorously at restrictive temperature on plates for at least 20 generations, as they formed thick, wild-type-like colonies and had a plating efficiency of 100% in these growth conditions (Fig. 1C and D). Second, the seven suppressed strains did not form filaments after 2–4 h of incubation at high temperature while *dnaE*(Ts) strains did (as illustrated for the *dnaE2.*6 and dnaE2*.6 pykAJP* strains Fig. 1E). Third, 4/4 spontaneously isolated suppressed strains (*dnaE2.4 pgkEP, dnaE2.4 pgmIP*, *dnaE2.4 pykAJP* and *dnaE2.6 pgm8*) grew as fast as the corresponding metabolic mutants at restrictive temperature (results with the *dnaE2.6 pgm8* strains are shown Fig. 1F).

**Figure 2:**
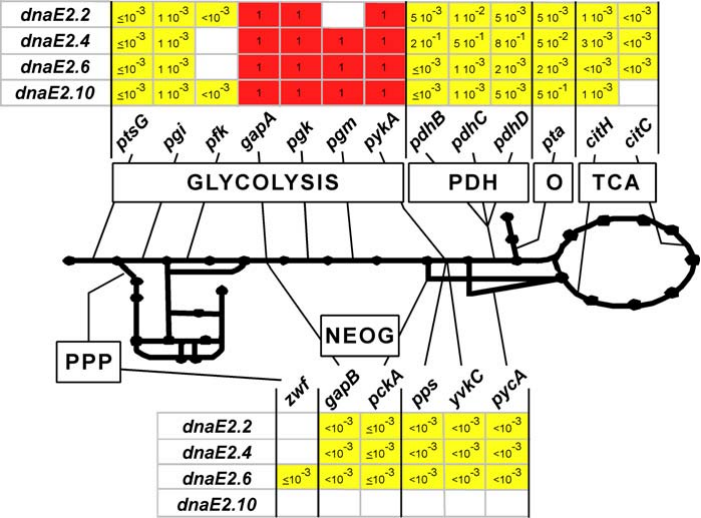
Suppressive mutations of *dnaE*(Ts) strains are clustered in the terminal reactions of glycolysis. To analyze the impact of metabolic mutations on the Ts character of *dnaE*(Ts) strains, the plating efficiency of double mutants at restrictive temperature was compared to that at permissive temperature in LB (boxed numbers). The level of filamentation was also analyzed for Tr strains. At least three independently constructed double mutants were tested. Metabolic mutations were deletions except for *pfk*, *pgk* and *pgm* were point mutations were used (*pfk1*, *pgkEP* and *pgm8*). Red boxes: strains fully suppressed (cells had a relative plating efficiency of 1, formed thick colonies, grew as fast as the corresponding metabolic mutant and did not make filament in liquid at restrictive temperature); yellow boxes: strains not suppressed (cells had a low plating efficiency at high temperature or, as in the *dnaE2.6 Δpdh* and *dnaE2.10 Δpta* strains, had a plating efficiency close to 1 but formed thin colonies with filamentous cells); white boxes: not tested. Thick lines and dots represent the central carbon metabolism reactions and their products, respectively. Main pathways are indicated. PDH: pyruvate dehydrogenase; O: overflow pathway; TCA: tricarboxylic acid cycle; PPP: pentose phosphate pathway; NEOG: gluconeogenesis.

To determine whether glycolytic mutations can suppress different *dnaE*(Ts) alleles and to search for other suppressive mutations, three of the above suppressors (*pgkEP*, *pgm8* and *ΔpykA*) and 16 other metabolic mutations (mainly deletions) in genes of the central carbon metabolism (see Fig. 2 for a schematic representation of the central carbon metabolism and inactivated genes) were introduced into the four *dnaE*(Ts) strains. Control experiments showed that none of the metabolic mutations affect viability of DnaE WT cells at high temperature in LB. Most metabolic mutations (15/19) were not suppressive as they did not restore cell viability on plates at high temperature (Fig. 2, yellow boxes). However, mutations mapping in the three-carbon part of glycolysis were all suppressive (red boxes). Consistently, the corresponding 15 double mutants had a plating efficiency of 100%, formed thick, wild-type colonies and were not filamentous in LB broth at high temperature (the lack of filamentation of the *dnaE2.6 ΔgapA* strain is illustrated Fig. 1E–F). Moreover, 4/4 newly constructed double mutants grew like the corresponding metabolic mutants at restrictive temperature (as shown for strains *dnaE2.6 ΔgapA*, *dnaE2.6 pgkEP* and *dnaE2.6 ΔpykA*, Fig. 1F).

**Figure 3:**
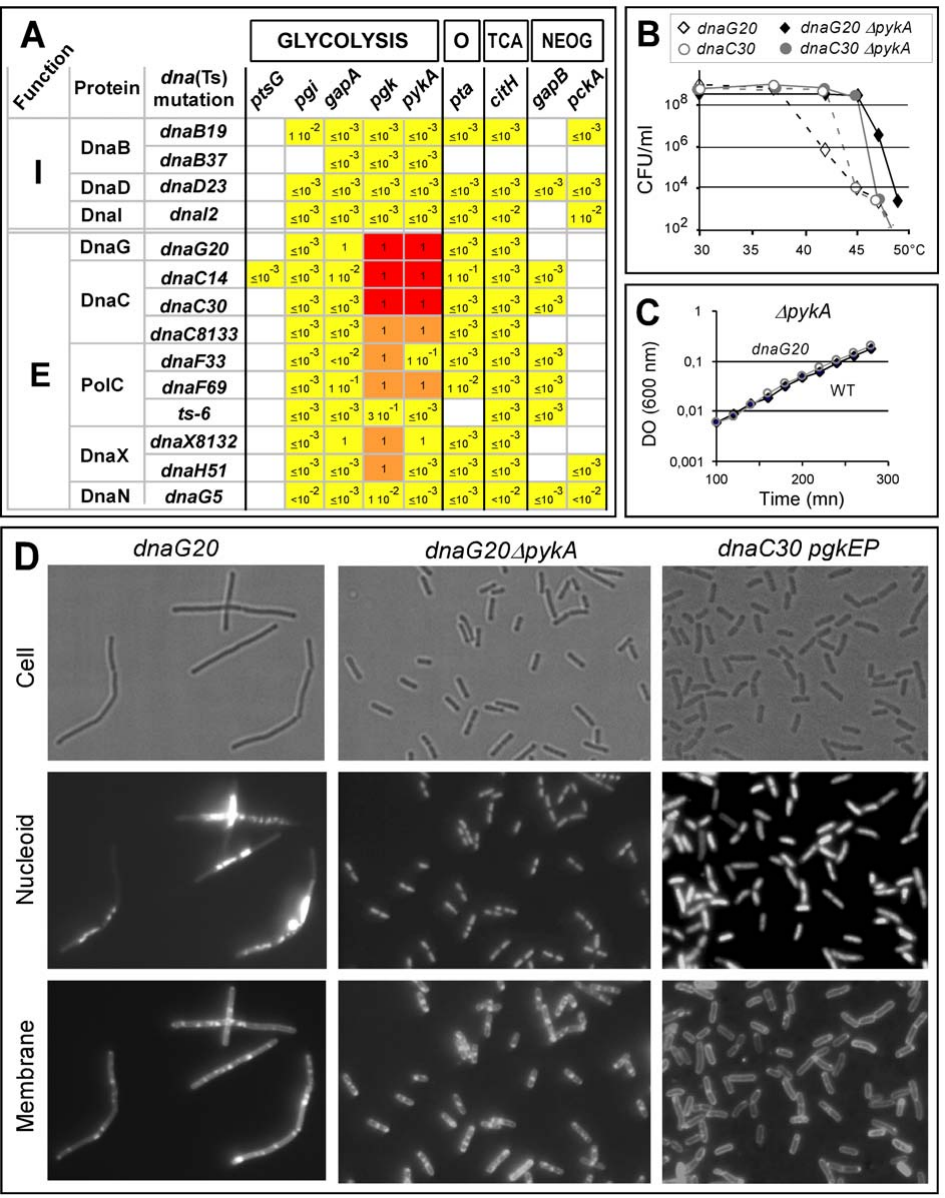
Mutations in terminal reactions of glycolysis can suppress *dnaG*(Ts) and *dnaC*(Ts) mutants. (A) The effect of 9 metabolic mutations on Ts mutations carried by initiation (I) or elongation (E) factors of DNA replication was investigated by the plating and filamentation assays. Relative plating efficiencies (see legend to Fig. 2) are indicated inside the boxes. All the tested metabolic mutations were deletion except in the case of *pgk* where the point *pgkEP* mutation was used. At least two independent double mutants were tested. Red boxes: strains fully suppressed (cells had a plating efficiency of 100%, formed thick colonies and are not filamentous in liquid at restrictive temperature); orange boxes: strains partially suppressed (cells had a plating efficiency of 100%, formed thick colonies but were filamentous in liquid at high temperature); yellow boxes: strains not suppressed (cells had a low plating efficiency at high temperature or, in a few instances, had a plating efficiency close to 1 but formed thin colonies containing filamentous cells); white boxes: not tested. (B) Plating analysis of Ts and suppressed strains at various temperatures. (C) Growth analysis of *dnaG20 ΔpykA* and *ΔpykA* mutants at 42°C in LB liquid browth. (D) Optical microscopy analysis at restrictive temperature of Ts and suppressed mutants (see legend of Fig. 1).

To further circumscribe the glycolysis/replication relationship, the effect of 9 metabolic mutations on 14 Ts mutations mapping in initiation and elongation replication genes was assessed (93 strains were tested). The effect of *ΔpykA* on two Ts division genes (*div104* and *ts1*) was also investigated. Only the mutations in the three-carbon part of glycolysis were suppressive. Full suppression (i.e. on plates and in liquid) was detected for three new *dna*(Ts) mutations mapping in the elongation factors DnaG (primase, *dnaG20*) and DnaC (helicase, *dnaC14* and *dnaC30*) (Fig. 3A, red boxes and Fig. B–D). Partial suppression (seen on plates but not in liquid) of one DnaC (*dnaC8133*), two PolC (*dnaF33* and *dnaF69*) and two DnaX (*dnaX8132* and *dnaH51*) mutants was also observed in some strains (the reason for different suppression efficiency on plates and liquid is not fully understood) (Fig. 3A, orange boxes). The remaining cells were not suppressed (Fig. 3A, yellow boxes). Taken together, the results establish a tight link between the three-carbon part of glycolysis and DNA replication. The robust suppression phenotype of some *dna*(Ts) mutations suggest that the link is particularly strong with the DnaE, DnaG and DnaC enzymes.

### Identification of metabolic alterations causing suppression

**Figure 4:**
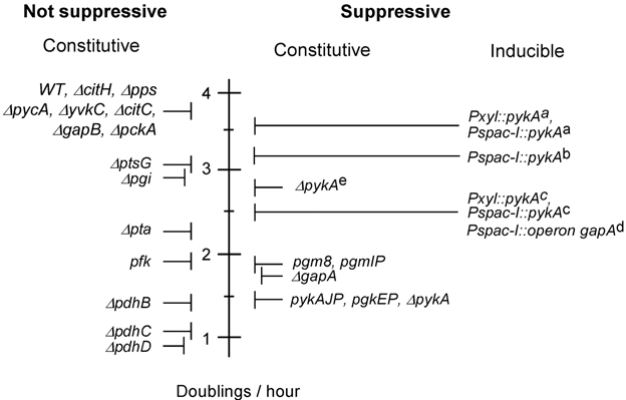
Suppression does not depend on a growth rate decrease. The doubling rate of metabolic mutants, WT for replication functions, was determined at 40–44°C by measurement of optical density of LB cultures. Values are positioned along a vertical axis. Mutations are grouped into suppressive, not suppressive, constitutive and inducible mutations. The growth rate of cells expressing glycolytic genes from the *Pspac-I* or *Pxyl* inducible promoters was positioned at the highest value allowing suppression for the following Ts alleles: ^a^: *dnaC30*; ^b and d^: *dnaE2.6*; ^c^: *dnaG20*. ^e^: Suppression of *dnaG20* by the *ΔpykA* mutation in the gluconeogenic medium CM (see Fig. 5 and the text below).

Mutations in genes of the central carbon metabolism often reduce cell growth. To test whether this reduction correlated with suppression, the doubling time of 20 metabolic mutants, WT for replication functions, was measured at high temperature in LB. A large range of growth rates, extending from the optimal value (∼3.8 doublings per hour) to a significantly lower rate (∼1 doubling per hour) was observed (Fig. 4). Suppressive mutations (*pgm8*, *pgmIP*, *ΔgapA*, *pgkEP*, *pykAJP* and *ΔpykA*) had a moderate growth rate (1.5–2 doublings per hour). Interestingly, some non suppressive mutations grew at a similar (*pfk, ΔpdhB*) or even lower (∼1 doubling per hour, *ΔpdhC* and *ΔpdhD*) rate. Moreover, suppression was observed in fast growing cells (2.5–3.5 doublings per hour, Fig. 4). These cells expressed glycolytic genes from inducible promoters (*Psapc-I* and *Pxyl*) or carried the *ΔpykA* mutation and were grown in a gluconeogenic medium (see below). Altogether, these data suggest that a mere decrease in growth rate is not a prerequisite for suppression.

Mutations in genes of the central carbon metabolism cause unscheduled alterations in concentration of metabolites and/or energetic compounds. Because (i) unbalanced metabolism might be sensed by cells as a stress, (ii) some nutritional stresses interfere with DNA replication [14], [15] and (iii) osmotic shocks can suppress Ts mutations in division and replication genes [29]–[31], the effect of various stresses on the Ts phenotype of replication mutants was assessed. For this purpose, the thermosensitivity of the *dnaE2.2, dnaE2.4, dnaE2.6, dnaG20* and *dnaC30* strains, WT for metabolic functions, was assessed on LB plates supplemented (separately or in combination) with chemicals causing osmotic (2% NaCl), energetic (125–500 µg/ml sodium azide) or nutritional (0.5 and 2 mg/ml DL-norvaline or 250 µg/ml arginine hydroxamate) stresses. In no case was suppression observed. Control experiments showed that, at the concentration used, the chemicals alter growth of WT cells but not their viability and plating efficiency. In case of arginine hydroxamate, WT cells did not survive to a 2 times higher concentration of the drug. This suggests that stress-like responses are not key determinants in suppression.

**Figure 5:**
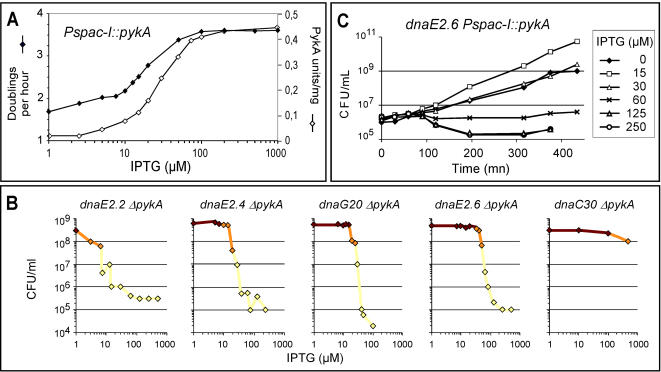
Suppression occurs abruptly at discrete and contrasted levels of PykA activity. (A) Modulation of PykA activity and growth rate. A WT strain expressing *pykA* from the IPTG inducible *spac-I* promoter was cultivated at 45°C in LB supplemented with various concentrations of IPTG (µM). The number of doublings per hour (closed diamonds) was determined from optical density measurements. PykA activity was measured from crude extracts prepared from cells growing exponentially in LB broth supplemented with various IPTG concentrations (open diamonds). Typical experiments are presented. (B) Abrupt phenotypic shift of *dna*(Ts) *Pspac-I::pykA* strains on plates. Strains were spread on LB plates containing various IPTG concentrations (µM). The number of colony forming units was counted after plate incubation at restrictive temperature. Red, yellow and orange symbols pinpoints IPTG concentrations where cells were Tr (forming thick colonies at high frequency), Ts (low plating efficiency) or shifting between the two phenotypes (variable plating efficiency and thin, heterogeneous colonies), respectively. (C) Abrupt phenotypic shift of the *dnaE2.6* *Pspac-I::pykA* strain in liquid. The strain was grown at 30°C in LB broth supplemented with various concentrations of IPTG (µM). Exponentially growing cells were then shifted to restrictive temperature (time 0) and maintained at low concentration (OD_600 nm_ <0.4) by serial dilutions when required. Aliquots were withdrawn at different times and the number of viable cells was determined by growing cells on plates containing 100 µM IPTG at 30°C.

To further identify metabolic alterations required for suppression, strains expressing a gene (*pgk* or *pykA*) or a group of genes (the whole *gapA* operon, which includes the suppressive *gapA*, *pgk*, *pgm* and *eno* genes) of the bottom part of glycolysis under the control of an inducible promoter were constructed. As glycolytic enzymes are abundant in living cells, we first tested whether the selected promoters (*Pspac-I* - IPTG dependent - or *Pxyl* - xylose dependent) were strong enough to modulate gene expression and hence glycolysis throughout a substantial range at high temperature in LB. Results with the *Pspac-I::pykA* strain (WT for replication functions) showed a significant modulation of PykA activity (from about 0.04 to 0.45 unit per mg of protein) (Fig. 5A). However, the modulation did not cover the whole range of values observed with the *ΔpykA* (0.006 unit/mg) and wild-type PykA+ (2.03 units/mg) strains, presumably because of some leakage and insufficient strength of *Pspac-I* (the weak increase in PykA activity and possibly growth rate at 0–15 µM IPTG could result from a non linear response of the *Pspac-I* promoter at low IPTG concentrations). Despite this limitation, *Pspac-I* allowed a growth rate modulation extending from that of the PykA+ and *ΔpykA* strains (compare Fig. 4 and 5A). Similar effects on growth rate were observed with the *gapA* operon expressed from *Pspac-I* and with *pgk* or *pykA* expressed from *Pxyl* (not shown). This shows that the inducible promoters are strong enough to substantially modulate cell metabolism.

To evaluate the impact of *pykA* expression on the Ts character of the *dnaE2.6* mutation, fresh *dnaE2.6 Pspac-I::pykA* cells grown at 30°C were spread on plates containing various IPTG concentrations and grown at restrictive temperature. Colony counting showed an abrupt shift from the Ts to Tr phenotype between 60 and 30 µM IPTG (Fig. 5B). This shift was also observed in liquid culture. Cells grew at ≤30 µM but not at ≥60 µM IPTG (Fig. 5C). They were filamentous at ≥60 µM but not at <15 µM (not shown). Interestingly, the Ts/Tr shift occurred at a PykA activity (0.35–0.4 unit/mg) allowing a growth rate (∼3.1 doublings per hour) which is only ∼15% lower than the optimal value (Fig. 4 and 5A). A similar abrupt shift in thermosensitivity of the *dnaE2.6* mutation was observed at a high growth rate when the whole *gapA* operon was expressed from *Pspac-I* or when *pykA* or *pgk* were expressed from *Pxyl* (not shown). Taken together, these data suggest that the Ts/Tr shift of the *dnaE2.6* strain occurs abruptly upon a moderate reduction (∼2 times) in expression of the genes encoding enzymes of the bottom part of glycolysis.

The *Pspac-I::pykA* fusion was then introduced in two other DnaETs mutants (*dnaE2.2* and *dnaE2.4*), one DnaC mutant (*dnaC30*) and in the *dnaG20* strain and suppression was assessed on plates as above. All mutants shifted from the Ts to Tr phenotype upon modulation of PykA expression (Fig. 5B). In the range of linear response of promoter activity to inducer concentration (about 10–100 µM), the shift again occurred in a narrow (∼2) window of IPTG concentration. Interestingly, despite the similar window size, the Ts mutants underwent the phenotypic shift at drastically different levels of PykA expression and growth rate. For instance, the *dnaE2.2* mutant shifted at low PykA concentration and growth rate while *dnaE2.6* and *dnaC30* shifted at high PykA concentrations and growth rates (Fig. 4). It is inferred from this that Ts mutants shift at discrete, specific and highly diverse levels of expression of genes of the three-carbon part of glycolysis. These results further support the notion that growth rate is not a key determinant in suppression.

LB is a complex rich medium that likely contains glycolytic and gluconeogenic carbon sources. It however confers a glycolytic regimen to exponentially growing cells as mutants deleted for the glycolytic *pykA* or *gapA* genes grew at a slower (∼2.5 times) rate than WT cells or strains deleted for the gluconeogenic *pckA* or *gapB* genes (Fig. 4). To further characterize the metabolic regimen (glycolytic or gluconeogenic) in suppressive and non suppressive contexts, the activity of the *gapB* promoter, an indicator of gluconeogenesis, in the WT strain and in metabolic mutants was measured. Using *lacZ* transcriptional fusion, it was previously shown that *gapB* expression is repressed (≤20 β-Galactosidase units) in glycolytic carbon sources and strongly induced (≥200 β-Galactosidase units) on gluconeogenic nutrients [32]. Using this construction, we observed a glycolytic regimen for both suppressive (*ΔpykA* and *pgkEP*) and non suppressive (WT, *Δpgi* and *ΔpdhC*) contexts (Table 3). This suggests that the suppression phenotype observed above occurs in a glycolytic regimen.

**Table 3:** Suppression in LB occurred in glycolytic regimen

Genetic context	Suppressive activity	β-Gal activity§
WT	No	5.6+/−5.3
*Δpgi*	No	14.7+/−15.5
*pgkEP*	Yes	4.4+/−0.7
*ΔpykA*	Yes	1.9+/−1.4
*ΔpdhC*	No	4.6+/−10.3

§ The activity of the *gapB* promoter fused to *lacZ* was measured in WT, suppressive and non suppressive contexts. Values correspond to β-galactosidase activity (Miller units/mg of total protein) with standard deviation. All the tested strains exhibited a high β-galactosidase activity (∼200 units/mg) when grown in gluconeogenic conditions.

In order to test whether suppression can also take place in gluconeogenesis, suppression was assessed in CM, a minimal medium supplemented with gluconeogenic carbon sources (casein hydrolysate and malate) that allows a high growth rate (∼2.9 doublings per hour at 46°C) to WT cells. This medium is gluconeogenic as mutations in the three-carbon part of the gluconeogenesis pathway (including the reversible glycolytic reactions *pgm* and *pgk* and the gluconeogenic genes *pckA* and *gapB*) prevented (or strongly reduced) growth in CM, while inactivation of the glycolytic PykA enzyme had no effect on growth rate (not shown). The plating assay showed that *dnaE2.2*, *dnaE2.4*, *dnaE2.6*, *dnaG20* and *dnaC30* strains, WT for metabolic functions, were Ts in CM. The isogenic *ΔpykA* strains were all but one (the *dnaC30 ΔpykA* strain) Tr on plates. Taken together, these data showed that suppression of some Ts mutations can occur in the neoglucogenic regimen.

### Impact of metabolic alterations on DnaTs protein activity

Suppression could have resulted from changes in the replisome that make Ts proteins dispensable for DNA synthesis. Two sets of data showed that this is not the case. First, most of the suppressed strains exhibited a residual thermosensitivity at extreme *B. subtilis* growth temperatures (cf Fig. 1D and 3B). Second, disruption of the *dnaE* gene in strains carrying a suppressive mutation was not possible unless the recipient strain carried an additional copy of the gene (not shown). Hence, survival at high temperature of suppressed strains requires at least partially active Ts proteins.

**Figure 6:**
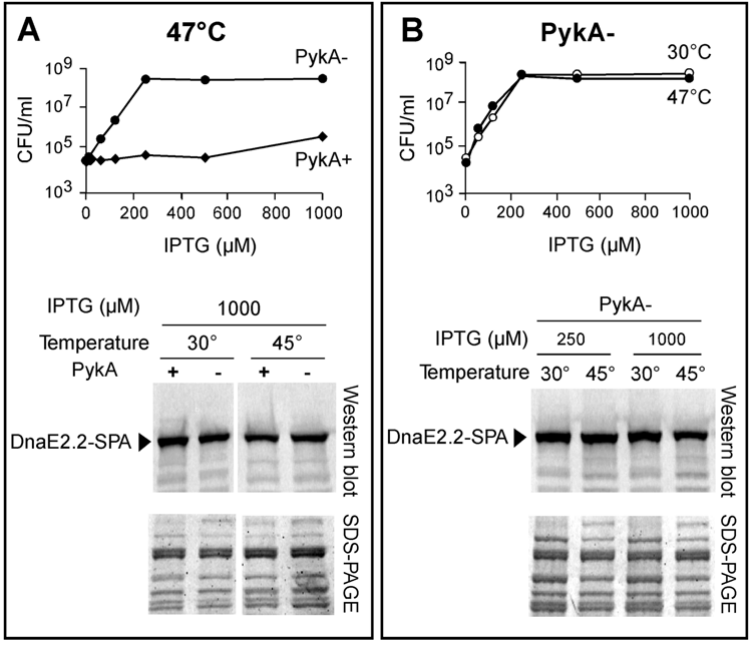
Suppression is due to maintenance of DnaTs protein activity at high temperature. (A) Suppression is not due to DnaETs protein accumulation. Top panel: Plating efficiency of *PspacI::dnaE2.2-SPA* PykA+ and PykA− cells plated on medium supplemented with various IPTG concentrations and incubated at 47°C (restrictive temperature on plates). Bottom panel: SDS-PAGE (stained with SYPRO-Red for total protein staining) and Western blot analysis of crude extracts (10 µg of total proteins) prepared from cells grown ∼5 generations in 1000 µM IPTG at 30 or 45°C (restrictive temperature in liquid cultures). (B) The DnaETs proteins are Tr in the PykA− context. Top panel: Plating efficiency of the *PspacI::dnaE2.2-SPA ΔpykA* strain grown in various IPTG concentrations at 30 and 47°C. Bottom panel: SDS-PAGE and Western blot analysis of crude extracts prepared from cells grown ∼5 generations at 30 or 45°C in 250 or 1000 µM IPTG.

Transcriptional fusions of the *dnaE*, *dnaC* and *polC* promoters with the *lacZ* reporter gene indicated that suppression does not result from *dna*(Ts) genes over-expression (Table 4). In order to test whether it might result from Ts protein accumulation, a set of PykA+ and PykA- strains encoding from *Pspac-I* the DnaE-2.2, -2.4 or –2.6 Ts proteins fused to the SPA tag [33] at the C-terminus was constructed. The plating assay showed that the *dnaE*(Ts)-*SPA* PykA+ cells were Ts and that the isogenic PykA− strains were Tr at high IPTG concentrations (as illustrated in the top panel of Fig. 6A with the *dnaE2.2-SPA* allele). This suggested that DnaETs-SPA proteins respond to glycolytic mutations like the original DnaETs proteins. Despite the contrasted phenotype of the PykA+ and PykA− strains, both cell types contained similar amount of DnaETs-SPA fusion proteins at saturating amount of IPTG (1000 µM) as shown by Western blot analysis with anti-SPA antibodies (Fig. 6A, bottom panel). Moreover, the *dnaE2.2-SPA* *ΔpykA* strain exhibited a similar IPTG dependence and intracellular DnaETs-SPA concentration at permissive and restrictive temperature (Fig. 6B). Similar results were observed with the *dnaE2.4-SPA* *ΔpykA* strain (not shown). Taken together, these results suggest that (i) suppression does not depend on Ts protein accumulation and that (ii) the DnaTs proteins are Tr in the PykA− background.

**Table 4:** Suppressive mutations do not stimulate promoters of replication genes

Promoter	Genetic context§	
	PykA+	PykA−
*PdnaE*	4.3+/−2.2	6.9+/−1.3
*PdnaC*	52+/−18	41+/−21
*PpolC*	11+/−2	11+/−2.3

§ Promoter activity was measured in PykA+ and PykA− strains carrying (i) the *lacZ* ORF downstream of the promoter of *dnaE*, *dnaC* or *polC* and (ii) the replication genes under the control of *Pspac-I*. The β-galactosidase activity was measured in cells growing exponentially at 37°C in LB containing 1 mM IPTG (in these growth conditions, the IPTG-driven expression of *dnaE*, *dnaC* and *polC* has no effect on cell growth and morphology [5], [93], [96]. Values correspond to Miller units/mg.

## Discussion

**Figure 7:**
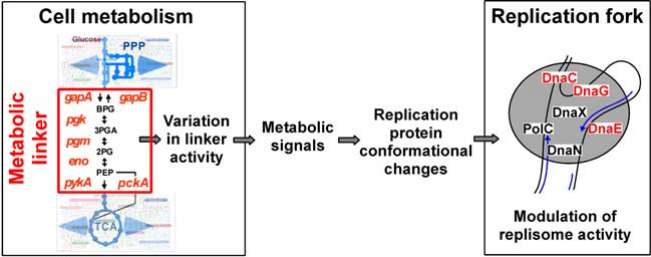
Model of glycolysis and replication connection. Signals generated according to the activity of the metabolic linker cause conformational changes in replication proteins to modulate replisome properties. The thick blue arrows pinpoint main sites of carbon diffusion.

DNA replication and carbon metabolism are fundamental to life. Here, we report a robust genetic link between them. This link, discovered during an extensive analysis of *dna*(Ts) suppressors, is very strong as some metabolic alterations fully restore growth of *dna*(Ts) mutants at a temperature at which a lethal arrest of DNA synthesis otherwise occurs. Two major lines of evidence show that this link is highly specific in terms of both replication and metabolism. In the case of replication, full viability is restored to mutants affected in 3 DNA elongation factors out of 9 replication functions tested (3 specific for DNA initiation and 6 mainly involved in DNA elongation). These factors are the lagging strand polymerase DnaE, the DnaC helicase and the DnaG primase. These enzymes probably act on the same DNA strand (the lagging strand template) in the replication fork and two of them (DnaG and DnaC) interact [5], [7], [34]. In the case of metabolism, full viability is restored only by mutations in the 5 successive terminal reactions of the three-carbon part of glycolysis/gluconeogenesis out of 21 mutations leading to inactivity or absence of enzymes of the central carbon metabolism. Interestingly, two lines of observations suggest that these 5 terminal reactions are at a key position in the overall metabolism of the cell. First, these reactions provide a single route for the catabolism of nutrients (via both glycolysis and gluconeogenesis) and form therefore one of the most active parts of the metabolic network in any growth conditions. Second, the enzymes involved are highly abundant and conserved in the three kingdoms of life [35], [36]. For these reasons, the three-carbon part of the glycolysis/gluconeogenesis pathway can be viewed as an ancestral linker that organizes the metabolic pathway network into two domains including, in one case, the upper part of glycolysis and branched reactions, and, in the other case, the TCA cycle and branched reactions (Fig. 7, left panel). The linker might also serve non-metabolic roles, as its enzymes are often multifunctional and essential even in rich media containing nutrients that simultaneously enter the central carbon metabolism at various levels [35], [37]–[42]. Data reported here and elsewhere indicated that one of these new roles might be to coordinate principal cellular functions [21], [28], [40]–[49].

The molecular basis of the Ts phenotype of the *dnaE*, *dnaG* and *dnaC* mutants has not been fully analyzed. However, two mutants (*dnaE2.10* and *dnaE2.6*) are defective in the elongation phase of DNA replication as they cease incorporation of labeled precursors shortly after a shift to restrictive temperature (47°C) [5] (unpublished data). The *dnaE2.2* and *danE2.4* mutations are also considered to affect elongation, as cells carrying these mutations exhibit a strong decrease (albeit, not an arrest) in the rate of precursor incorporation (unpublished data) and form long filaments at restrictive temperature (in bacteria, the latter response is induced when perturbations in DNA chain elongation lead to generation of single-stranded DNA [50]). Cells containing *dnaG* and *dnaC* mutations also form filaments at restrictive temperature and are thus tentatively classified as elongation mutants as well. The likely high diversity in the processes leading to inhibition and rescue of replication in *dnaE*, *dnaG* and *dnaC* mutants contrasts sharply with the limited number of metabolic mutations, clustered in the three-carbon part of glycolysis, that lead to suppression. For this reason, we hypothesize that restoration of viability proceeds through a similar mechanism in all the suppressed strains.

Several hypotheses can account for the viability of *dna*(Ts) mutants at high temperature. In one hypothesis, growth restoration is due to DnaTs proteins being protected from heat-inactivation by a stress-like response induced by metabolic alterations. However, the following observations make this possibility unlikely. First, separate or combined induction of the general stress regulon and the stringent response by chemicals inducing nutritional, energetic or physical stresses was not suppressive. Second, efficient suppression is observed with Ts mutations occurring in only three replication proteins while a stress-like-induced mechanism would be expected to cause a more random profile of suppression because of the relaxed effect of thermoprotector agents on protein conformation [51]–[53]. Third, it is unexpected that thermoprotector agents would respond efficiently to numerous but sometimes limited changes in linker activity but not to any alteration elsewhere in central carbon metabolism.

A second hypothesis is that the restoration of viability of *dna*(Ts) cells results from *dna*(Ts) genes over-expression and DnaTs protein accumulation. This hypothesis is based on recent evidence that several metabolic enzymes (aconitase, TktA…) play a role in the organization and packaging of bacterial and mitonchondrial genomes, and thereby, can modulate DNA supercoiling and expression of genes with promoters sensitive to DNA topology [54], [55]. This hypothesis is rejected as neither *dna* gene over-expression (the expression of *dnaE* and *dnaC* was analysed) nor DnaTs protein accumulation (3 *dnaE*(Ts) alleles were tested) are detected in suppressed cells. Topological changes might also rescue cell growth by stimulating expression of genes involved in the stabilisation, repair or restart of altered replication fork [56], [57]. While attractive, this hypothesis is discarded as (i) promoters of the genes involved in these processes are not sensitive to DNA topology [54], [58] and (ii) it is unlikely that the stabilization, repair and restart pathways can rescue mutations that arrest DNA synthesis.

A third hypothesis relies on the role played by the ribonucleotide reductase (RNR) and the dNTP pool on DNA replication. The synthesis of dNTP depends on RNR that converts NDPs into dNDPs. In *E. coli*, transcription of the RNR encoding gene is stimulated during initiation of DNA synthesis and this stimulation is required for DNA elongation [59], [60]. Moreover, RNR would be part of a large dynamic structure that contains the replication machinery and the dNTP synthesis apparatus [61]–[63]. These functional and structural relationships between RNR, DNA elongation, and the replication machinery raise the possibility that RNR and/or the dNTP pool are involved in restoration of growth to Ts mutants. Obviously, this hypothesis makes sense in the case of mutations altering the polymerase DnaE. It however should not be involved in suppression of mutations in DnaC and DnaG whose activity (DNA unwinding and primer synthesis, respectively) depends on NTP but not on dNTP.

A fourth hypothesis is that the restoration of viability is the result of a change in the characteristics of initiation or of chain elongation or of both. In *E. coli*, mutations that restore viability to a thermosensitive DNA elongation mutant (*dnaX2016*) have previously been isolated [64], [65]. They map to *dnaA*, a gene that encodes the major initiator of DNA replication. During initiation, DnaA binds to short repeated sequences located in the chromosomal *oriC* region, partially unwinds *oriC*, and recruits the helicase. The helicase then interacts with the primase that synthesizes primers at which the replisome is assembled for initiating DNA synthesis [reviewed in [2], [10]]. It was found that the restoration of viability to *dnaX*(Ts) *dnaA* double mutants at high temperature does not result from a strong stimulation of DNA elongation. Indeed, in 3/3 suppressed strains analyzed at high temperature, DNA synthesis remained strongly disturbed, leading to a high proportion of filamentous cells containing an incorrectly compacted and distributed nucleoid [66]. The lack of effect on DNA elongation was further supported by the fact that restoration of cell viability was only observed at temperatures allowing residual DNA synthesis (i.e. suppression did not happen at temperatures inhibiting DNA synthesis). Instead of depending on elongation stimulation, restoration of viability of *dnaX dnaA* double mutants was shown to result from a decrease in the efficiency of initiation. This decrease is a direct consequence of *dnaA* suppressive mutations that significantly weaken the initiation activity of DnaA [66], [67]. It was thus proposed that these *dnaA* mutations restore viability at high temperature by reducing the number of chromosome per cell to a level that allows the partially active DnaX protein to support ongoing chromosomal replication and cell growth [66]. Survival of *E. coli* cells to mild inhibition of chain elongation by drugs also relies on mutations that reduce the efficiency of initiation [68]. In contrast, our results suggest that the restoration of viability to *B. subtilis dna*(Ts) cells results mainly from stimulation of DNA elongation rather than from modifications in initiation efficiency. First, suppression can occur at temperatures at which DNA synthesis is arrested. This is at least the case for the *dnaE2.10* allele which can be suppressed at temperatures ranging from 47–50°C while DNA synthesis is already arrested at 47°C in Ts cells WT for glycolytic genes [5]. Second, DNA elongation seems to be normal in all (21/21) the suppressed strains identified here, as, unlike the original *dna*(Ts) mutants, these cells do not form filament at high temperature and their nucleoid is properly compacted and distributed. It should also be noted that despite the relatively large study conducted here, no suppressive mutations mapping in any of the four initiation genes (*dnaA*, *dnaB*, *dnaD* and *dnaI*) were isolated despite the total size of the DNA in these potential target genes being similar to that of the suppressive glycolytic genes (4.4 versus 6.7 kb, respectively). These observations therefore suggest that the restoration of cell viability in *E. coli* and *B. subtilis* elongation mutants depends on different mechanisms and that in *B. subtilis*, this mechanism mainly operates via stimulation of DNA chain elongation.

Stimulation of DNA elongation in *B. subtilis* suppressed cells might result from changes in replisome requirements. One possibility is that there is a change in the composition of the replisome that allows replication to occur despite the absence of the DnaTs proteins. There is no precedent for this, most likely because the function of DnaC, DnaG and DnaE (duplex unwinding, primer synthesis and lagging strand polymerization, respectively) are essential for DNA synthesis and cannot be carried out by other replisomal proteins. Nevertheless, they might theoretically be ensured by alternative replication factors as those encoded, for example, by prophages. While attractive, this possibility can be discounted because (i) studies on dnaE(Ts) mutants have all been carried out in cells devoid of the three main *B. subtilis* prophages (SPβ, PBSX and Skin) [69]and (ii) control experiments showed that the restoration of viability of *dnaC*(Ts) and *dnaG*(Ts) mutants, initially observed in prophage-containing cells, occurs with the same efficiency in prophage-free strains (not shown). It is also discarded that the putative alternative replication factors are of chromosomal origin since *B. subtilis* does not encode structural and/or functional homologues of the DnaC helicase and DnaG primase that could replace efficiently these proteins at the fork [70]. In contrast to these hypothesis, two observations suggest that restoration of viability of *dna*(Ts) cells requires DnaTs proteins. First, most of the suppressed strains are thermosensitive at extreme temperatures. Second, the *dnaE* gene cannot be inactivated in cells carrying a suppressive mutation. Hence, restoration of viability cannot be explained satisfactorily by changes in the composition of the replisome.

An alternative possibility for stimulating DNA elongation in *dna*(Ts) cells is a functional change in the replisome, a hypothesis consistent with all our results. Such a functional change might be due to suppressive metabolic mutations either making some activities of DnaTs proteins dispensable for DNA synthesis or protecting these activities from heat-inactivation. It may therefore be significant that (i) suppressed and non-suppressed strains contain the same amount of DnaETs protein at restrictive temperature and (ii) the minimal DnaETs concentration required for survival of (PykA−) suppressive strains is similar at permissive and restrictive temperature. As mentioned above, DnaC, DnaG and DnaE have catalytic activities that are essential for DNA synthesis and cannot be efficiently ensured by alternative proteins. Their heat inactivation can thus only be countered by a process allowing their thermo-protection. However, replication enzymes might also be required at the fork for structural and/or regulatory purposes. If the loss of these additional functions could be tolerated in certain conditions, this loss might be suppressed not only by thermo-protection but also by processes making these functions dispensable at the fork. The function(s) defective in DnaTs proteins at high temperature need to be identified to further dissect the molecular basis of stimulation of DNA elongation in suppressed cells. However, irrespective of the detailed mechanism, an attractive hypothesis is that suppression involves conformational changes in replisomal enzymes.

Our physiological studies identify the activity of the metabolic linker region as important in restoration of viability. This restoration occurs abruptly over a narrow range of metabolic linker activity and the absolute value of this range varies greatly with the Ts allele in question. However, viability can be restored without major change of the growth rate or the direction of carbon flow through the metabolic linker. The absence of effect of reversing the carbon flow rules out catabolite repression, a genetic system that regulates expression of numerous genes in highly energetic glycolytic carbon sources [71], as a major factor in suppression. Moreover, restoration of viability cannot be due to a reduction in ATP concentration since (i) inhibition of ATP synthesis is not suppressive and (ii) ATP is present at a constant level over a large range of metabolic activities and growth rates (from 2.3 to <0.4 doublings per hour) [72]–[74]. We therefore propose that the postulated conformational changes in replisomal enzymes of suppressed cells depends on changes in the activity of the metabolic linker.

Our suppression assay provides the first evidence for a genetic system that connects DNA chain elongation to glycolysis. Its role in WT cells may be to modulate some aspects of DNA synthesis in response to the provided energy. The molecular mechanism of the linker effect on the postulated conformational changes in replisomal enzymes remains to be elucidated. We viewed the system as comprising the metabolic linker, a signal-generating activity of this linker, and signal-mediated conformational changes in replisomal proteins, possibly the lagging-strand DnaE polymerase, DnaC helicase and DnaG primase (Fig. 7). These conformational changes might result from protein binding to metabolites, post-translational modifications and/or protein-protein interactions. Metabolite binding (mainly ATP) and post-translational modifications (mainly phosphorylation) of replication proteins are frequently reported in the literature (see for instance) [8], [75]–[78]. Examples of physical interactions between replication proteins and metabolic enzymes are on the contrary scarce. However, interactions between the primase DnaG and subunits of the pyruvate dehydrogenase, an enzyme that converts the glycolysis product (pyruvate) into acetyl-CoA, have been detected in *B. subtilis* using the yeast two hybrid technology [34]. Moreover, evidence was also obtained in both prokaryotes and eukaryotes for an interaction between the replication machinery and a complex involved in dNTP synthesis. This interaction is proposed to form a dynamic hyperstructure in which a high concentration of dNTP is provided to support the very high rate (∼700 nucleotide/s) of DNA synthesis in bacteria [61], [79]. It is also significant that glycolytic enzymes can be part of non-metabolic protein complexes that may integrate central carbon metabolism and various cellular functions (see for instance [28], [43], [44], [49], [80]). Moreover, at least three enzymes of the metabolic linker seem to be endowed with a protein kinase activity (GapA, Pgk and PykA) [42], [45], [47] (our unpublished observations). This later observation raises the possibility that the activity of the metabolic linker regulates the conformation of replication fork proteins by phosphorylation. Unexpected and fundamental regulation via phosphorylation by a metabolic linker enzyme has recently been shown in human cells, in which nervous flux can be regulated by the GAPDH-driven ATP-dependent phosphorylation of the GABA_A_ neuronal receptor [47].

Several reports in the literature suggest that the link between DNA replication and cell metabolism is ubiquitous (see above citations). Understanding the underlying mechanisms is thus of general interest. Such studies might also be of medical importance as early events in carcinogenesis, which generally include up-regulation of glycolysis (the Warburg effect) and a decrease in DNA stability and replication fidelity [81], [82], might involve perturbations of the replication/metabolism link.

## Materials and Methods

### Strain construction and growth conditions

*B. subtilis* strains are listed in Supplementary Table S1, S2, S3. The *E. coli* strain used for plasmid constructions was DH5α (*supE*44 *supF*58 *hsdS*3(r_B_^−^m_B_^−^) *dapD*8 *lacY*1 *glnV*44 *Δ*(*gal-uvrB*)47 *tyrT*58 *gyrA*29 *tonA*53 *Δ*(*thyA*57)). *B. subtilis* strains carrying the *dna*(Ts) mutations in a prophage-free background (DGRM1-4) were constructed by transforming TF8A competent cells with total DNA of EDJ strains. Transformants were selected at 30°C on phleomycin (Pm) containing plates (the phleomycin gene - *pmr* - is located just upstream of *dnaE*) and the presence of the *dna*(Ts) mutation was checked by analyzing cell growth and filamentation at restrictive temperature by plating and optical microscopy analysis. To construct *dnaH51*, *dnaC8133* and *ts-6* prophage-free strains (DGRM12, 13 and 266, respectively), a similar strategy was used except that a Trp+ DNA was mixed to the transforming *dna*(Ts) DNA. Transformants were first selected for Trp+ at 30°C and then toothpicked to select Ts colonies (note that the procedure cured the spectinomycin (Sp) marker of BD54 ts-6). To construct strains (WT for DNA replication functions) lacking Zwf, Pps or PycA metabolic enzymes (DGRM34, 35 and 38), the corresponding genes, cloned into an *E. coli* vector, were interrupted by an erythromycin (Em) cassette. The mutations were then introduced in the *B. subtilis* genome by transforming TF8A cells with the plasmid cut in the vector backbone. Strains resulting from a double crossover event were selected by PCR. Metabolic mutations carried by strains provided by different laboratories were transferred in the TF8A context using transformation and appropriate marker selection (DGRM14-17, 25, 27–33, 36 and 37). To label spontaneously isolated suppressors mapping in genes of the *gapA* operon, the spectinomycin marker (*spc*) was inserted downstream of the operon (in *araE*) by transforming spontaneously suppressed strains (DGRM5, 6, 8–11) with a PCR product composed of *spc* flanked by the 5′ and 3′ region of *araE*. The yielded strains were named DGRM61, 62, 64, 83, 102 and 105. To label the *pykAJP* suppressive mutation, the corresponding suppressed strain (DGRM7) was transformed to erythromycin resistance with DNA of the BFS67 strains (from the European collection of *B. subtilis* mutants [83]) carrying an EmR determinant (*ery*) in the vicinity of *pykA*. The presence of the PykA mutation in a representative transformant (DGRM65) was confirmed by showing that the constructed strain grew as slowly as the parental strain in LB and in minimal medium supplemented with glucose. To evaluate the impact of spontaneously isolated suppressive mutations on metabolic enzyme activity, suppressive mutations labeled by the *spc* or *ery* markers were transferred into the WT TF8A strain by transformation yielding strains DGRM18–24. Cell slow growth in LB at 45°C was used as a criteria for the presence of the metabolic mutation in the produced strains. A large (∼200) collection of strains carrying a metabolic mutation and a replication or division Ts mutation (apart the spontaneously isolated strains) was generated by transforming Ts cells with total DNA extracted from strains containing a labeled metabolic mutation. The genotype of the constructed strains was checked by analyzing their Ts at 51°C (the maximal growth temperature of *B. subtilis*), growth rate at 30°C (metabolic mutants generally grow slower than WT strains in LB), back-cross studies, PCR analysis and/or Southern blotting. To construct strains (WT or Ts for replication functions) expressing PykA from the IPTG inducible promoter *spac-I*, a segment corresponding to the 5′ region of *pykA* (extending from position −42 to +412 according to the first base of the ORF) was generated by PCR and cloned between the *Hind*III-*Bam*HI sites of the *E. coli* (Em^R^) plasmid pMUTIN2 [84]. Once validated by DNA sequencing, the yielded circular plasmid was introduced upstream of the chromosomal *pykA* gene of TF8A. This puts *pykA* under the control of *Pspac-I*. The resulted strain (DGRM26) was verified by PCR and by analyzing growth rate and PykA activity as a function of IPTG concentration. The fusion was then transferred to various *dna*(Ts) strains yielding DGRM260–265. For complementation studies, WT copies of the *pgk*, *pgm*, *eno* or *pykA* genes were PCR amplified and cloned downstream of the xylose inducible promoter *pxyl* of plasmid pAX01, at the *Bam*HI site [85]. In the generated plasmids (checked by DNA sequencing), the transcriptional fusion is included in a cassette labeled by the *spc* marker and delimited by the front and back regions of the *B. subtilis* *lacA* locus. The labeled fusion was then introduced at the *lacA* locus of the WT (TF8A) strain by double crossover. The structure of the yielded strains (DGRM242–246) was controlled by PCR. The construction what then introduced by transformation into strains carrying a labeled glycolytic mutation or into *dnaE*(Ts) strains mutated in a glycolytic gene (DGRM247–259). Conditional expression of the metabolic genes was checked by following cell growth in LB complemented or not with 0.5% xylose. To measure the effect of metabolic mutations on carbon flux polarity, competent cells of the GM1514 strain that contains *lacZ* downstream from the *gapB* promoter at the *amyE* locus were transformed with the total DNA extracted from strains carrying metabolic mutations (168 Pgi::spc, 501–77, GM1501, GTD040 and DGRM18, 25). Transformants were selected on plates supplements with appropriate antibiotics. The presence of the metabolic mutations and of the fusion were verified by analyzing cell growth in LB at 37°C and by PCR, respectively. For strains carrying the *pgkEP* mutation, the presence of a functional *PgapB::lacZ* fusion was verified by measuring β-galactosidase activity upon transfer of the structure in the 168 strain. The constructed strains were termed DGRM239–241 and 285. To measure replication promoter activity in suppressed and non suppressed strains, we introduced by transformation the *ΔpykA::spc* mutation (from strain DGRM25) into strains carrying the *PdnaE::-*, *PpolC::-* or *PdnaC::lacZ* fusions and β-galactosidase activity was measured in the parental PykA+ (HVS597, 607 and 614) and yielded PykA− (DGRM322–324) strains. To investigate whether suppression depends on accumulation of DnaETs proteins, we first constructed PykA+ or PykA− strains carrying the *dnaE*(Ts) genes under the control of *Pspac-I*. For this, we transformed the *dnaE*(Ts) *pmr* strains (DGRM1-3) with the DNA extracted from cells carrying the *Pspac-I-dnaE* *ery* construction (HVS614). Transformants were selected at 30°C on plates containing EmR and 500 µM IPTG. A toothpicking analysis on 100–200 transformants allowed us to identify Ts EmR PmS cells. Growth analysis at 30°C (turbidity and optical microscopy analysis) in liquid and solid rich medium supplemented or not with 500 µM IPTG showed that the selected strains containas expected *dnaE* under the control of *Pspac-I*. The strains were termed DGRM230–232. To construct the isogenic PykA- strains, competent *spac-I-dnaE*(Ts) cells were transformed to SpR by the DNA extracted from the *pykA::spc* strain (DGRM25). Representative IPTG-dependent, slow growing EmR transformants were selected (DGRM233–235). That these cells carry the *dnaE*(Ts) mutation was controlled by transferring the EmR region into the 168 strain and showing that most of the transformants were Ts. We then fused the DnaETs proteins to the SPA tag [33]. For this, competent cells of the constructed strains were transformed with the total DNA extracted from a strain (FLB5) containing the *dnaE-SPA* fusion labeled with a *neo* marker. A Western blot analysis of KmR transformants was carried out using the anti-SPA monoclonal anti-FLAG M2 antibody (Sigma, St Louis, MO). Clones encoding an IPTG inducible fusion protein of the expected size were thus identified. Among these, a genetic screen allowed the selection of representative strains containing the *dnaE*(Ts) mutation. The genetic analysis indicated that the SPA fusion decreases the activity of the DnaETs proteins as the corresponding cells were more IPTG dependent than the isogenic counterpart lacking the fusion. The structure of the *dnaE* region in the constructed strains (DGRM304–306 and 308–310) was checked by PCR. Plasmids used in the above mentioned constructions are available upon request.

Cells were grown in LB or minimal medium supplemented with glucose 0.2% or malate 0.5% [86]. MM-CM is composed of minimal medium supplemented with caseinhydrolysate and malate (0,2% each). In case of strains carrying the *pta* mutation, acetate (1%) was added to LB in order to improve cell growth and prevent appearance of fast growing variants (control experiments showed that this complementation does not interfere with suppression). The *ΔgapA* strain was grown in the presence of 1 mM IPTG in order to allow *Pspac*-I-driven expression of the metabolic genes located downstream of *gapA* [87]. To induce the general stress regulon and/or the stringent response, NaCl (2%), sodium azide (125–250 µM), DL-norvaline (0.5 or 2 mg/ml) and/or arginine hydroxamate (250 µM) were added separately or in combination to LB as previously described [15], [88]–[90]. *B. subtilis* and *E. coli* competent cells were prepared as previously described and antibiotics were added to the media at regular concentrations [86], [91].

### Isolation and localization of spontaneously isolated suppressive mutations

In order to search for suppressive mutations, four *dnaE*(Ts) strains (DGRM1–4) cured of the three major prophages (SPβ, PBSX and SKIN) were used. Tr derivatives were selected at the lowest temperature reducing 10^4^–10^5^ fold the platting efficiency of the Ts mutants (see below the temperature used). For each mutant, 20–40 cultures of 5 mL of LB were inoculated with 10^4^ cells and grown at 30°C under shaking. At saturation, 10^6^ to 10^7^ cells were spread on LB plates and incubated at restrictive temperature. After 48–72 hours of growth, a large and a small colony from each plated culture were selected and streaked for further growth at restrictive temperature. An isolated colony was then cultivated at 30°C in liquid LB and stocked at −80°C. To identify strains carrying an extragenic suppressor, chromosomal DNA of Tr variants was extracted and used to transform the WT 168 strain to Pm^R^. Twenty to fifty transformants selected at 30°C were then toothpicked on Pm plates and grown at high or low temperature. Suppressive mutations were assumed to be extragenic when the linkage between the Tr marker and *pmr* was ≤85%. This procedure allowed us to identify 29 independent extragenic suppressors.

To precisely map the suppressor (*JP*) carried by strain DGRM7 and located in the vicinity to *dnaE2.4* (genetic linkage of ∼80%), a collection of cells carrying an Em^R^ marker at various positions in the *dnaE* region was used (strains of the Bacillus functional analysis - BFA -, see Supplementary Table S2). The DNA of these cells was used to transform competent cells of the suppressed *dnaE2.4 JP* strain. Transformants were selected on Em + Pm plates at 30°C. For each transformation, the proportion of Tr colonies among 50 Em^R^ Pm^R^ transformants was determined. The highest Em^R^-*JP* linkage was observed with strains BFA93 (60%) and BFA91 (45%) suggesting that the *JP* mutation maps ∼5 kb downstream of *dnaE*. This was confirmed by transforming the original *dnaE2.4* strain to thermoresistance with PCR fragments generated from the suppressed strain and containing overlapping sequences downstream of *dnaE*. DNA sequencing of the region revealed a 81 bp deletion in the central part of the glycolytic *pykA* gene. This mutation was termed *pykAJP*.

Mapping of *sup8*, a *dnaE2.6* suppressor unlinked to *dnaE* (strain DGRM9), was carried out by random transposition as described previously [92]. To enrich for cells carrying the transposon (*Tn*) in the vicinity to *sup8*, DNA of a large pool of cells that had suffered transposition was extracted, used to transform the parental *dnaE2.6* strain to Cm^R^ (the *Tn* marker) at 30°C and Cm^R^ Tr transformants were selected by replica-plating. This second generation Cm^R^ Tr cells was then pooled and processed as above for further enrichment of *Tn*-*sup8* linked genomes. Genetic linkage analysis showed that a representative Cm^R^ Tr strain carried *Tn* in the vicinity of *sup8* (linkage of 24%). To further map *Tn*, the DNA of this strain was restricted with several enzymes, run on agarose gel along with DNA ladders, and analyzed by Southern blot using as a probe a ^32^P labeled DNA homologous to *Tn*. The size of the *Tn*-containing segments was then compared to the predicted restriction map of the *B. subtilis* genome using a software developed in the laboratory (available upon request). This allowed us to map *Tn* in a 585 bp segment located between coordinates 3,444 862 to 3,445 447 of the *B. subtilis* genome. This location was confirmed by PCR. To map *sup8*, mutants of the BFA collection carrying the Em^R^ determinant in the *Tn* region were used (see Supplementary Table 2). The highest Em^R^-*sup8* linkage (90%) was observed with strain BFA1079 which carries the Em^R^ determinant in *yvbK*. DNA sequencing of the region revealed a single mutation (termed *pgm8*) in the glycolytic *pgm* gene, ∼8 kb downstream of *yvbK*.

In order to determine whether additional suppressive mutations map in genes of the central carbon metabolism, the genetic linkage between suppressors and an Em^R^ marker located in the vicinity of metabolic genes in BFA mutants (see Supplementary Table S2) was investigated. For this, competent cells of 5 suppressed strains (DGRM5, 6, 8, 10 and 11) were transformed to Em^R^ at 30°C with DNA of BFA cells. The proportion of Ts/Tr transformants was then determined by toothpicking 20–50 colonies on selective plates incubated at permissive or restrictive temperature. Surprisingly, all the suppressive mutations were located nearby *yvbK* like *sup8* (linkage of 60–90%). As the suppressed strains grew poorly on minimal medium supplemented with glucose, it was speculated that they carry a mutation in the glycolytic genes located downstream from *yvbK*. Consistently, single mutations were found by sequencing in *pgk* (*pgkEP*), *pgm* (*pgmIP* and *pgm25*) and *eno* (*enoLP*). All the suppressive mutations are described in Table 2.

### Restrictive temperatures for *dna*(Ts) mutants

Restrictive temperatures, defined as the lowest temperatures reducing 10^4^–10^5^ fold the plating efficiency of Ts mutants on LB (for the plating assay) or reducing 10^1^–10^3^ cell viability after 2.5 hours of incubation in LB broth (for the filamentation assay), were as follows (P: plating assay; F: filamentation assay): *dnaE2.2* and *dnaE2.4* (P: 47°C, F: 44°C); *dnaE2.6* (P: 42°C; F: 40°C); *dnaE2.10* (P: 49°C; F: 47°C); *dnaG20* (P: 45°C; F: 42°C); *dnaC14* (P: 49°C: F: 45°C); *dnaC30* (P: 45°C; F: 43°C); *dnaC8133* (P: 47°C; F: 45°C); *dnaX8132* (P: 45°C; F: 45°C); *dnaF33* (P: 37°C); *dnaF69* (P: 42°C); *dnaH51, ts-6*, *dnaB37* and *dnaD23* (P: 45°C); *dnaG5* and *dnaI2* (P: 47°C). For studies carried out in MM-CM, selective temperatures were as above except for *dnaE2.2* (P: 45°C; F: 42°C) and *dnaE2.4* (P: 47°C; F: 42°C).

### DNA/protein manipulations and enzymatic assays

DNA extraction and manipulations were carried out according to standard procedures [86], [91]. DNA sequencing was carried out on PCR products with the PRISM Sequencing Kit and the 377A sequencing apparatus from Applied Biosystem (Warrington) according to the manufacturer recommendation. Western blot analysis of DnaE-SPA production, β-Galactosidase activity and the amylase assay were carried out as previously described [83], [93]. Protein concentrations were determined with a Bio-Rad Protein Assay using BSA as a standard. β-Galactosidase is expressed in Miller units per mg of protein. PykA activity was measured by employing the linked lactic dehydrogenase assay, as modified [94], [95]. Crude extracts were prepared as follows: cells from 25 ml cultures at OD_600_ = 0.3 were centrifuged, resuspended in 75 µl of lysis buffer (60 mM Na_2_HPO_4_, 40 mM NaH_2_PO_4_, 10 mM KCl, 1 mM MgSO_4_, 1mM DTT, 0.1 mg/ml lysozyme, 0.01 mg/ml Dnase), incubated 20 mn on ice, 5 mn at 37°C, 15 mn at 20°C, centrifuged 10 mn at 12000 g and the supernantant was stored at −20°C. The standard assay system contained in 1 ml: 50 mM Imidazole HCl pH 7.1, 10 mM MgCl_2_, 50 mM KCl, 5 mM ADP, 5 mM phosphoenolpyruvate, 0.2 mM NADH, 10 units lactate dehydrogenase. Reactions were initiated by the addition of 0.5 to 10 µl of crude extract in a 1-cm quartz cuvette, and kinetics of NADH oxidation was followed at 340 nm in a Lambda 20 Perkin Elmer spectrophotometer at 25°C. One unit of enzyme is defined as the amount required to convert 1 µmole of phosphoenolpyruvate to pyruvate per min in the standard assay.

### Microscopy analysis

To check for cell filamentation, parental Ts strains incubated 2–3 hours at restrictive temperature and suppressed strains grown for at least 5 generations were stained 5 mn in the presence of 4′,6′-diamidino-2-phenylindole (DAPI) and FM5-95 (Molecular Probes) for visualizing the nucleoid and membrane, respectively. They were then deposited on glass slides covered with 1.2% agarose in minimal medium, and examined with a Leica (Leica DMRA2, Leica Microsystems GmbH, Wetzlar, Nl) microscope with a 100× magnification oil-immersion objective and a CDD camera (Photometrics CoolSNAP HQ, Roper Scientific Inc, Duluth, GA). Cell pictures were captured with METAMORPH V5.0 (Universal Imaging, Media, PA, USA).

## Supporting Information

Table S1 (0.05 MB XLS)

Table S2 (0.03 MB XLS)

Table S3 (0.05 MB XLS)
